# Novel Bioinformatics Approaches for Analysis of High-Throughput Biological Data

**DOI:** 10.1155/2014/814092

**Published:** 2014-12-28

**Authors:** Julia Tzu-Ya Weng, Li-Ching Wu, Wen-Chi Chang, Tzu-Hao Chang, Tatsuya Akutsu, Tzong-Yi Lee

**Affiliations:** ^1^Department of Computer Science and Engineering, Yuan Ze University, Taoyuan 320, Taiwan; ^2^Innovation Center for Big Data and Digital Convergence, Yuan Ze University, Taoyuan 320, Taiwan; ^3^Institute of Systems Biology and Bioinformatics, National Central University, Taoyuan 320, Taiwan; ^4^Institute of Tropical Plant Sciences, National Cheng Kung University, Tainan 701, Taiwan; ^5^Graduate Institute of Biomedical Informatics, Taipei Medical University, Taipei 110, Taiwan; ^6^Bioinformatics Center, Institute for Chemical Research, Kyoto University, Kyoto 611-0011, Japan

## 1. Introduction

With the advent of high-throughput technologies, molecular biology is experiencing a surge in both growth and scope. As the amount of experimental data increases, the demand for the development of ways to analyze these results also increases. For example, the next-generation sequencing (NGS) technology has generated various sequencing data. Mass spectrometry- (MS-) based experiments are also widely applied in proteomics studies. Rapidly advancing technologies have offered us the opportunities to examine the genome, transcriptome, and proteome in comprehensive ways. Yet, extracting meaningful information from this vast sea of data and approaching biological problems from systems biology perspective have become the Holy Grail in bioinformatics. The main focus of this special issue is novelty: new ideas, original research findings, and practical applications that intend to answer biological questions through high-throughput technologies. The papers in this special issue present methods and experiments that demonstrate novel platforms and systems and new bioinformatics tools and models, as well as new data-analytical methods for high-throughput biological data.

In this special issue, U. Rosani et al. attempted to unravel the genome of* Mytilus galloprovincialis*, the Mediterranean mussel, through a target capture and high-throughput massive sequencing approach to reduce whole genome sequencing cost and effort. However, inferences from sequencing data rely heavily on careful experimental design, as well as efficient detection and removal of artifacts. While analyzing restriction-based reduced representation genomic data, D. C. Ilut et al. demonstrated that, by setting an optimal clustering threshold, false homozygosity or heterozygosity can be effectively minimized.

With the advancement of genomic researches, the number of sequences processed in comparative methods has grown immensely. E. A. Marucci et al. developed a parallel algorithm for multiple sequence similarities calculation using the k-mers counting method. Their tests showed that the algorithm provides a very good scalability and a nearly linear speedup. In “*A de novo genome assembly algorithm for repeats and nonrepeats*,”  Z. Dai et al. proposed a new genome assembly algorithm called the sliding window assembler (SWA), which assembles repeats and nonrepeats by adopting a new overlapping extension strategy to extend each seed and implementing a compensational mechanism for low coverage datasets. Results of their analysis on three datasets support the practicability and efficiency of SWA as a promising algorithm for NGS data.

High-throughput technology holds great promises for the efficient investigation of transcriptomes, but the enormous amount of gene expression data demands effective analytical tools. By combining three gene-set analytical methods in one R statistical package, C.-Y. Chien et al. presented MAVTgsa, offering a systematic pipeline for the identification of significant gene-set modules from a set of gene expression data. Often, genes are coexpressed and coregulated or interact together to orchestrate a series of biological processes. To decipher the complex genetic networks associated with different cellular functions, M. Huerta et al. proposed to study the expression dependence between not only coexpressed genes but also sets of coexpressed genes.

In an attempt to predict the survival time in patients with oral squamous cell carcinoma, O. Hamidi et al. demonstrated that the three sparse variable selection techniques, when applied on gene expression microarray data, were able to yield better prediction results. For bladder cancer, Y.-H. Wong et al. proposed a statistical method based on carcinogenesis relevance values (CRVs) to identify 152 and 50 significant proteins and subsequently generated novel protein-protein interaction (PPI) network markers for early and late stage bladder cancer. Their findings not only provide new clues specific to cancer but also offer cancer researchers new directions for targeted cancer therapy.

In metagenomics, C.-M. Chiu et al. developed a pipeline for the systematic analysis of the association between gut flora and obesity through high-throughput sequencing and bioinformatics approaches. Eighty-one stool samples were collected and the V4 region of 16S rRNA genes was selected for metagenomics analysis. The results demonstrate that bacterial communities in the gut could be clustered into the N-like (normal) group and OB-like (obese) group. Remarkably, most of the normal samples were clustered in the N-like group, and the OB-like group was enriched with case samples, indicating that bacterial communities in the gut were highly associated with obesity. The results provide new insights into the correlation of gut flora with the rising trend in obesity.

In order to explore the molecular mechanism of flounder sex determination and development, Z. Fan et al. applied RNA-seq technology to investigate the transcriptomes of flounder gonads, obtaining 22,253,217 and 19,777,841 qualified reads from the ovary and testes, respectively. These reads were jointly assembled into 97,233 contigs. Among them, 2,193 contigs were identified to be differentially expressed in the ovary and 887 in the testes. Following annotation, several sex-related biological pathways including ovarian steroidogenesis and estrogen signaling pathways were revealed in the flounder for the first time.

Several bioinformatics tools are now being employed to analyze high-throughput expression data. In an attempt to study the molecular changes as a result of radiation exposure, K.-F. Lee et al. designed a set of expression microarray experiments studying the changes in gene and microRNA expression in peripheral mononuclear blood cells treated with varying doses of radiation. Combined with the existing tools for biochip analysis, K.-F. Lee et al. identified the various pathways associated with the exposure to differing doses of radiation and the potential gene-microRNA interactions that regulate these pathway changes.

The rapid increase in microRNA NGS data demands the development of comprehensive and customized tools for data analysis. In “*Large-scale investigation of human TF-miRNA relations based on coexpression profiles*,” C.-H. Chien et al. developed a computational strategy to investigate the transcription factors of human miRNA genes on a global scale. The proposed method helps enhance our understanding of the transcriptional regulatory mechanisms of miRNAs. On the other hand, in “*miRSeq: a user-friendly standalone toolkit for sequencing quality evaluation and miRNA profiling*,” C.-T. Pan et al. introduced a new tool for NGS data alignment that not only is easy to implement but also offers various methods for evaluating sequencing quality and provides profiles for up to 105 species for users to compare with. These studies demonstrate that customizability, easy access, and user-friendliness are crucial to high-throughput data analysis.

In the analysis of protein catalytic sites, C.-S. Yu et al. found that the side chain of catalytic residues usually points to the center of the catalytic site. The results demonstrate that the proposed method (EXIA2) could outperform the existing methods on several benchmark datasets that include over 1,200 enzyme structures. In “*High-throughput functional screening of steroid substrates with wild-type and chimeric P450 enzymes*,” P. Urban et al. identified the structural features of steroid-based substrates catalyzed by CYP1A enzymes containing wild-type and synthetic variants. The results are interesting and may help extend the scope of knowledge surrounding the structural properties of enzymes recognizing and metabolizing exo- and endogenous substrates including drugs. In “*Bioinformatic prediction of WSSV-host protein-protein interaction*,” Z. Sun et al. used bioinformatics methods to identify possible protein-protein interactions between white spot syndrome virus (WSSV) and its shrimp host. Their findings provide certain insights to readers in the relevant fields. In “*MPINet: metabolite pathway identification via coupling of global metabolite network structure and metabolomic profile*,” F. Li et al. demonstrate a network-based metabolite pathway identification method, which identifies novel pathways related to disease.

In clinical medicine, length of stay (LOS) in the intensive care unit (ICU) of spontaneous intracerebral hemorrhage (sICH) patients is one of the most important issues. C.-L. Chan et al. showed that the threshold of a prolonged ICU stay is a good indicator of hospital utilization in ICH patients. This indicator can be improved using quality control methods such as complications prevention and efficiency of ICU bed management. Patients' stay in ICUs and in hospitals will be shorter if integrated care systems are established. In “*Ultrasonographic fetal growth charts: an informatic approach by quantitative analysis of the impact of ethnicity on diagnoses based on a preliminary report on Salentinian population*,” A. Tinelli et al. provide customized fetal growth charts to formulate an accurate fetal assessment and to avoid unnecessary obstetric interventions. The fetal growth assessment is crucial to the health of newborns. Results suggest a careful reexamination for the appropriateness of continued use of currently adopted reference growth curves to classify neonates.

This special issue presents novel applications or methodologies of biomedical or bioinformatics analysis. The selected articles show the importance of integrating diverse ideas and multidisciplinary knowledge to answer complex biological questions through high-throughput technologies.

